# Artificial cells and biomimicry cells: A rising star in the fight against cancer

**DOI:** 10.1016/j.mtbio.2025.101723

**Published:** 2025-04-03

**Authors:** Renata Faria Maia, Asma Sadat Vaziri, Mohammad-Ali Shahbazi, Hélder A. Santos

**Affiliations:** Department of Biomaterials and Biomedical Technology, The Personalized Medicine Research Institute (PRECISION), The University Medical Center Groningen (UMCG), University of Groningen, the Netherlands

**Keywords:** Artificial cell, Precision medicine, Nanotechnology, Cancer therapy, Drug delivery

## Abstract

Biomimetic Artificial Cells (ACs) are engineered systems that mimic the properties and functions of natural cells, offering significant potential for biomedical applications. The performance and applicability of these synthetic constructs depend on the choice of materials and fabrication methods. Our review delves into the materials, fabrication techniques, and diverse applications of ACs, emphasizing their transformative impact on the field of cancer therapy as smart vehicles for drug delivery, immune system stimulation, cancer cell targeting to minimize off-target effects and maximizing therapeutic efficacy as well as *in vitro* models for cancer research. By providing a comprehensive overview, we aim to elucidate how these synthetic cells can move the field forward, offering innovative solutions to longstanding challenges in cancer treatment and opening new frontiers in less toxic treatment options.

## Graphical abstract

ACs can mimic the immune cells, target cancer cells, and function as models for cancer research. The review also explores challenges and future directions, including improve therapeutic efficacy, and develop less toxic treatment options. While ACs hold significant potential to revolutionize cancer therapy, substantial research is required to address existing drawbacks and fully harness their capabilities.

**Figure:**
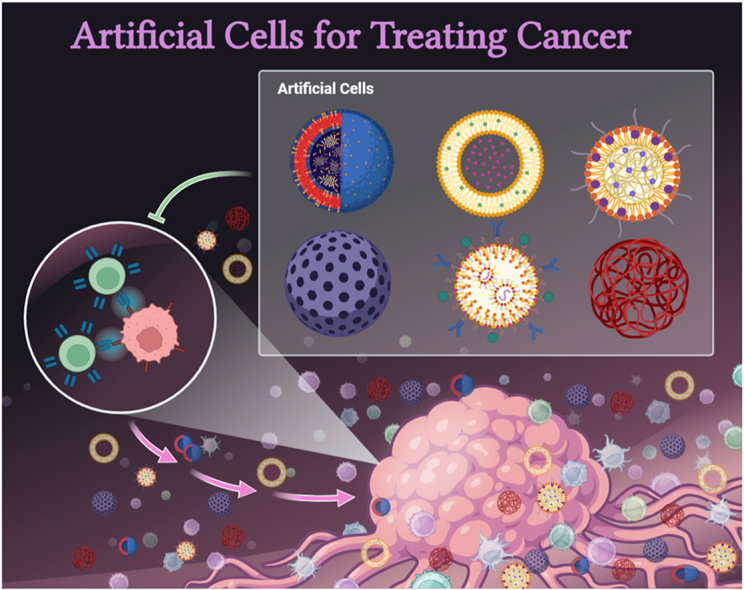

## 1 Introduction

Cancer continues to be one of the challenges in contemporary healthcare [1]. Despite significant advancements in conventional therapies, limitations like systemic toxicity, limited targeting accuracy, and drug resistance still remain [2]. Thus, innovative and multifaceted therapeutic strategies are crucial [3]. Artificial Cells (ACs) are promising contenders for revolutionizing cancer treatment [4] by replicating the properties and functions of cells [[5], [6], [7]]. ACs are engineered particles that mimic one or more functions of biological cells. They often consist of biological or polymeric membranes enclosing biologically active materials. These cells can be synthetically made to capture energy, maintain ion gradients, store information, and replicate molecular pathways. On the other hand, other type of systems as biomimicry cells can be used. Biomimicry cells are created by copying the strategies and designs found in nature to improve the delivery or targeting of the inspired system [8]. One example of biomimicry cells in cancer treatment involves cell membrane-coated biomimetic nanoparticles. These nanoparticles are designed to mimic the properties of natural cell membranes, allowing them to effectively target cancer cells while minimizing damage to healthy cell [9]. Artificial cells, protocells, vesicles and synthetic cells are very similar terms used in multidisciplinary of the fields. Synthetic cells, also known as ACs or minimal cells, are engineered particles that mimic one or many functions of biological cells. They often consist of biological or polymeric membranes enclosing biologically active materials. Protocells are self-organized, spherical collections of lipids that are considered rudimentary precursors to living cells. They are formed from abiotic components and display characteristics similar to biological cells, such as compartmentalization and primitive metabolic functions. Vesicles are small cellular compartments formed by a lipid bilayer that separates their contents from the cytoplasm or extracellular environment. They perform various functions, including transport, digestion, protection, secretion, and osmoregulation.

**Fig. 1:**
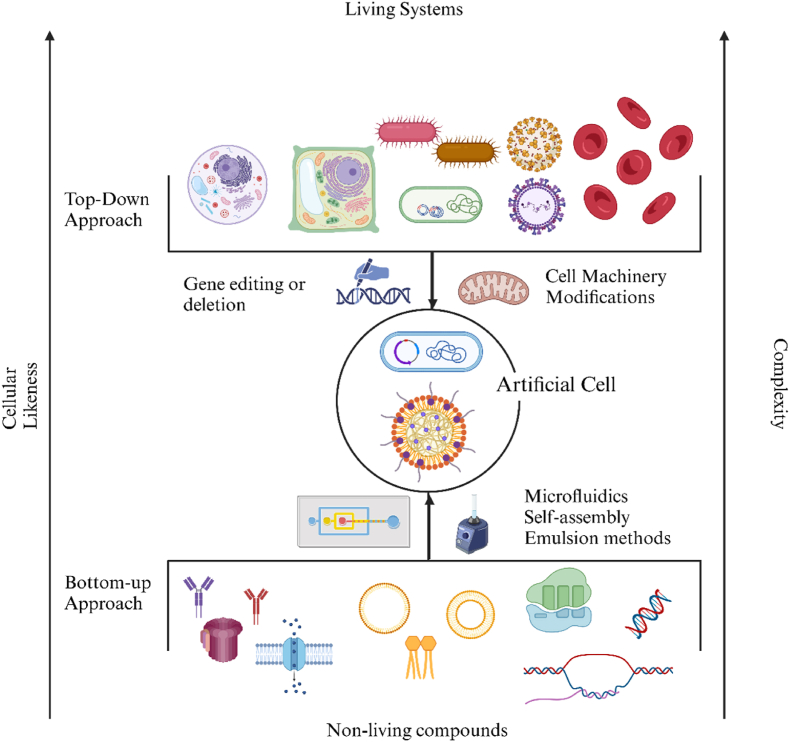
The two approaches of synthetic biology to form ACs: Top-down and bottom-up. Created by Biorender.

ACs can be applied as building blocks for molecular systems engineering, and host synthetic biology pathways. ACs are constructed using both top-down and bottom-up approaches, as shown in Fig. 1. The top-down approach aims to simplify living organisms by modifying their cellular machinery and metabolic pathways, or by introducing synthetic organelles or molecular systems to reprogram cell functions. This method also involves deleting unnecessary genes and regions in their genomes to target specific functions while preserving the natural structure. However, the gene editing techniques limited the practical applications of top-down methods [10]. Conversely, The bottom-up approach involves combining non-living and active compounds, such as lipids, polymers, natural cell membranes, and nanoparticles (NPs) to form cell-mimicking contracts via hybrid methods [[11], [12], [13], [14], [15], [16]]. Bottom-up ACs offer unparalleled control over design and functionality, enabling customization for specific tasks by incorporating proteins and genes into their surface or encapsulating inside of a cell [17].

The first stage in constructing a cellular model involves preparing a sealed container with a bilayer structure resembling the cellular membrane found in nature. Consequently, a boundary-like system is the most basic characteristic response that a cell can perform [18]. Among all the approaches to mimic cell membranes, lipid vesicles emerged as the starting point to construct an AC [19]. ACs made of lipids are more sophisticated constructs designed to replicate certain aspects of real cells for research or therapeutic purposes [[20], [21], [22], [23]]. They can be used to understand biological mechanics and investigate the dynamic interface of membranes [24,25]. The composition and complexity of ACs distinguish them from the more straightforward lipid vesicles [26]. Other materials can be employed to create ACs as Proteinosomes are made from protein-polymer conjugates. They can encapsulate biochemical reactions and mimic cellular processes as they are used in synthetic biology to create cell-like systems for studying biochemical pathways, drug delivery, and as microreactors for industrial processes [27]. Polymersomes, on the other hand, retain cargos more efficiently due to their thermodynamic inertness and low lateral diffusivity, which vary with the molecular weight of the polymer, hydrophobic block nature, and glass-transition temperature [28,29]. However, their low membrane permeability can be an issue for certain applications. Advanced polymersomes can be functionalized for tuneable permeability and lateral mobility, with some achieving similar properties to liposomes and cell membranes [30]. Hydrogels are ideal for providing a gel-like chassis for incorporating biological and nonbiological components in ACs, enabling the replication of cellular functionality [31,32]. Hybrid approaches, combining different materials, have gained attention for their potential to synergize the advantages of individual components.

**Fig. 2:**
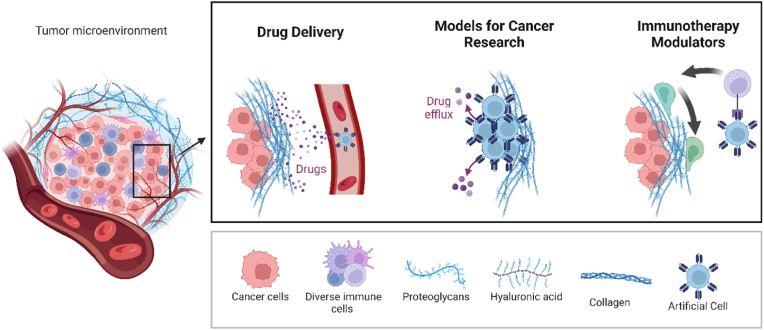
Diverse applications of ACs in cancer treatment and research. Created by Biorender.

In cancer, ACs can perform distinct functions as displayed in Fig. 2. ACs and biomimetic cells serve as advanced responsive delivery systems, encapsulating and transporting potent anti-cancer drugs directly to tumor sites. Their biocompatible design minimizes off-target effects and maximizes therapeutic efficacy. Also, ACs and biomimetic cells can be designed to mimic specific tumor microenvironments or natural cells, providing valuable tools for cancer research and drug development [33]. Therefore, it becomes imperative to understand the underlying principles of AC design, their interaction with the tumor microenvironment, and the potential impact on the efficacy and precision of cancer therapies. This review aims to provide a comprehensive overview of state-of-the-art research on ACs designed for cancer applications. Furthermore, we address the materials used for AC formation, fabrication methods, challenges, and future perspectives. And, we would take a board definition of ACs, which encompass a diverse range of structures and systems with nano and micro size.

## 2 Materials and properties of ACs

**Fig. 3:**
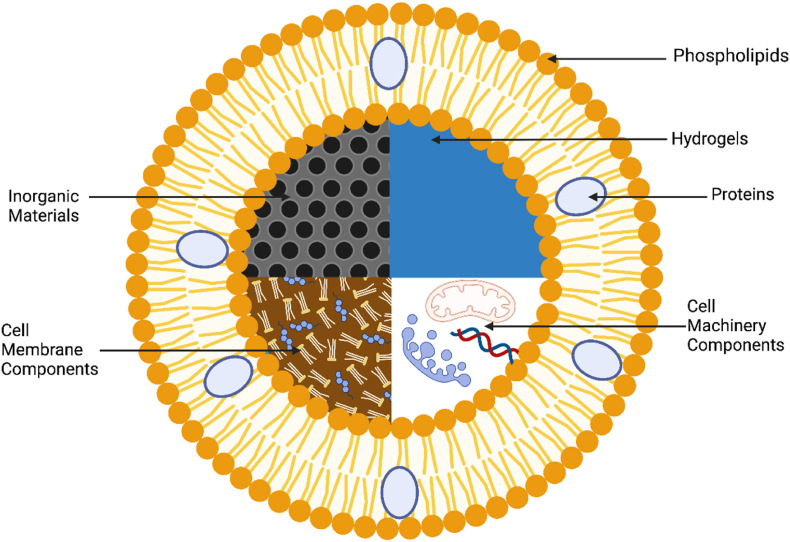
Distinct types of core materials used in ACs for cancer applications. The top-down approaches illustrated by a natural cell that can be edited. Lipid-based cores represent a widely explored phospholipids vesicles and the encapsulation of secondary lipid vesicles within a primary lipid vesicle creates a nested structure, often referred to as multi-compartment more closed to mimic a cell. Inorganic materials offer unique properties as their construction is represented in grey. Polymeric materials offer versatility represented by the large blue circle. Created by Biorender. (For interpretation of the references to color in this figure legend, the reader is referred to the Web version of this article.)

ACs are complex systems that require interdisciplinary collaboration for their development. At present, there is no universally accepted consensus on their classification or nomenclature [34]. The size of these systems is often used as a criteria for defining ACs. However, many Nano ACs are reported in the literature [25,35,36]. Therefore, does size matter? Size can influence their behaviour and functionality. However, without a universally accepted classification system, size is just one of many criteria researchers consider. The complexity and functionality of the micro/nano-particles are also crucial aspects to take into account. So why is it so difficult to classify them? Essentially, the multidisciplinary nature of the field means each discipline has its own terminology and classification preferences. Consequently, researchers use various criteria such as size, complexity, and functionality, leading to inconsistencies and complicating standardization efforts. Size can significantly influence the behaviour and functionality of ACs, making it a relevant criterion for classification. In this review, Micro ACs and Nano ACs were separated in order to better understand and underline the differences and similarities. One crucial aspect of constructing ACs is the choice of the core material. The chosen material must not harm surrounding tissues and cells. They often need to mimic specific cellular structures, like organelles, to perform desired functions. To illustrate the versatility of core materials, Fig. 3 summarizes the core materials employed in numerous studies with applications related to cancer.

Micro ACs are designed to mimic the functions of natural cells, which are employed as larger drug delivery systems, cell mimics for studying biological processes, and microreactors for biochemical processes [37]. In the cancer field, Micro ACs provide targeted drug delivery systems that can navigate the complex tumor microenvironment or being used as model to study drug permeability in tumor environment, as well as stimulate cells to present certain antigens. Microscale ACs typically range from a few micrometers to tens of micrometers (1–100 μm). These cells are constructed using materials like liposomes, polymeric nanoparticles, metal-organic frameworks (MOFs) and cell-derived materials. Each material offers distinct advantages, such as biocompatibility, controlled drug release, and high drug loading capacity, making them suitable for various therapeutic applications.

The idea of mimicking natural cell properties to enhance therapeutic efficacy has been a persistent goal. However, many current strategies do not focus on creating ACs that replicate the complex characteristics and size of natural cells. Instead of this, the idea is to biomimetic the structure of ACs or one single aspect of a living cell in a nano size, biomimetic ACs (nano ACs). Due to their smaller size, nano-ACs can penetrate tissues and cellular barriers more effectively than micro-ACs cells [38]. This makes them particularly useful for delivering therapeutics to hard-to-reach areas within the body. By utilizing materials like natural cell membranes, extracellular vesicles (EVs), genetically engineered cells, and synthetic biomimetic NPs, these cells can interact seamlessly with biological systems. The materials used to build Micro and Nano ACs are broadly categorized into core materials, membranes, and encapsulated systems. Table 1 highlights the diverse range of materials scientists are investigating for building micro ACs with applications in the cancer field.

**Table 1:** Different Materials used to build micro and nano ACs and their applications in the cancer field.

**Micro ACs**	**Type of approach**	**Core material**	**Membrane**	**Encapsulated systems**	**Method**	**Size of ACs**	**Application**	***Invitro***	***Invivo***	**Ref.**
	**Lipid-based**	Naturally, Derived L-A-Phosphatidylcholine (PC), L-A-Phosphatidylethanolamine (PE), Sphingomyelin (SM), And Cholesterol (CHOL)		Dox	Microfluidic Flow-Focusing Device	±100 μm	Biomimetic Tumour Environment	–	–	[39]
	**Cell-based**	Lymphocyte	Assembly Of peptides associated with major histocompatibility complex class (PMHC-I) and CD28 antibodies (αCD28)	–	Self-Assembly	10–20 μm	Drug Delivery	Splenocytes prepared from the spleen of OT-1 mice	C57 mice	[40]
	**Polymer -based**	Bovine Serum Albumin/Poly(*N*-Isopropylacrylamide) (BSA- NH2/PNIPAAM) Conjugate	–	Anti-Mir-223	Emulsion	2–5 μm	Drug Delivery	Human Primary Macrophages	Zebrafish (Danio)	[41]
		Hollow Polydopamine NPs	Bacterial Outer Membrane Vesicle and Cancer Cell Membrane (CCM)	–	Self-Assembly	180–600 nm	Drug Delivery	Melanoma cells (B16-F10 Cells), Normal human Dermal Fibroblasts (NHDFs) and Breast Cancer cell line (MCF-7) Cells	Delivery C57BL6 mice	[42]
		Alginate	–	Cell Suspensions Mixed With Streptavidin Solution	Microfluidics	120–180 μm	Biomimetic Tumour	Lung cancer cell line (A549), Collon Cancer cells (HCT116), Hepatocellular carcinoma cell line (HEPG2)	–	[43]
		Diethylaminoethyl(DEAE)-Dextran And PolyAcrylic acid (PAA)	–	Plasmid (Pcdna3.1-NOS3-3 (FLAG) Or Pegfp-C1)	Electrostatic Interaction-Mediated Liquid-Liquid Phase Separation	5.0 ± 1.4 μm PDI: 0.399	Drug Delivery	Hepatocarcinoma cells (SMMC-7721)	–	[44]
		Porous Silicon NPs	RBCs Functionalized With Cancer Antigen TRP2	–	Self-Assembly	634 ± 210 nm PDI: 0.55 ± 0.03	Immunotherapy Modulators	Peripheral blood mononuclear cells (PBMCs)	–	[45]
		Matrigel	CCM	mRNA siRNA	Emulsion	10–50 μm	Drug Delivery	Paclitaxel-resistance A549	BALB/c mice	[33]
**Nano ACs**	**Cell-based**	Mini-Simcells Simcells	–	Aspirin/Salicylate-Inducible Gene Circuit	Gene Editing	100–400 nm	Drug Delivery	Colorectal adenocarcinoma (Caco-2)	–	[46]
		NK	A Specific Targeting Aptamer (TLS11a)	–	Glycan Biosynthesis	78.7 ± 15.9 nm	Immunotherapy Modulators	HepG2	HepG2 Tumor-Bearing Mice (BALB/c mice)	[47]
		CCM		Histone and the Loaded DNA	Self-Assembly	31.8–116.1 nm PDI: 0.113.	Drug Delivery	HepG2, A549, Cervical cancer cell line (HeLa) and MCF-7 Cells	–	[48]
	**Polymer-based**	poly(lactic-co-glycolic acid)/(PLGA/DOTAP NPs)	Hybrid Membranes (Red Blood Cells (RBCs) And NCI-H1299 Cs)	SAHA	Double Emulsion	180–200 nm PDI: 0.3–0.4	Drug Delivery	Peripheral Blood Mononuclear Cells (PBMCs)	BALB/c mice	[49]
		poly(lactic acid) (PLA)	–	Complex Of Poly-[Haemoglobin-Tyrosinase]	Emulsion	–	Drug Delivery	B16F10 Melanoma Cell line	C57BL6 mice	[50]
		poly(ethylene glycol) diacrylate (PEGDA) NPs	RBC Membrane	DOX	Thin Film Hydratation Extrusion	120 nm	Drug Delivery	Breast cancer 4T1 Cells	BALB/c mice	[51]
	**Inorganic-based**	3D-Dendritic Mesoporous Silica NPs	PEG	DOX And Melittin	Emulsion	120 mm	Drug Delivery	MCF-7/Adr Dox-resistance Human Breast Cancer Cells	BALB/c mice	[52]
		Mesoporous Silica NPs	Chol	DOX And Indocyanine Green (ICG)	Emulsion	Around 220 mm	Drug Delivery	Pancreatic Adenocarcinoma Cell line (BxPC3)	Pancreatic Adenocarcinoma Cell line (BxPC3)	[53]
		Baso_4_ NPs	Transferrin	Zeolitic imidazolate framework (ZIF-8) Shell	Coprecipitation	323 ± 5 nm	Immunotherapy Modulators	4T1 Tumor cells and J774A.1 Macrophages	BALB/c mice	[54]
		Silica NPs	CCM	Deliver Chlorin E6 (Ce6)	Microemulsion	105 ± 5 nm	Drug Delivery	MCF-7 Cells	MCF-7 tumor-bearing mice model (BALB/c mice)	[55]
		Diatomite NPs	Anti-L1-Cell Adhesion Molecules (CAM)	Galunisertib	Microfluidic Co-Flow Device (Nanoprecipitation)	360 ± 50 nm PDI: 0.40 ± 0.05	Drug Delivery	Caco-2,colon cancer cell line resistant (HT29-MTX) and Colorectal cancer SW620 Cells	–	[56]

In Table 1, lipids are a cornerstone in the construction of ACs. They naturally form bilayer membranes, similar to those found in living cells, making them ideal for creating liposomes and other vesicle-based structures [57]. Synthetic lipid membranes are engineered to mimic the properties of natural cell membranes while offering enhanced stability and functionality. These membranes can be customized in composition, are more stable, and can incorporate functional molecules for targeted delivery [58]. Liposomes are widely use in cancer applications since 1995 with the approval of Doxil. Lipid NPs and Liposomes can enhance drug delivery to tumor sites through mechanisms like the enhanced permeability and retention (EPR) effect. On the other hand, lipid nanoparticles can be less stable than polymer-based systems, requiring careful formulation to prevent degradation.

Other commonly used material is polymers which include polylactic acid (PLA), polyglycolic acid (PGA), and their copolymers (PLGA) [59]. These materials are biodegradable, customizable in terms of mechanical properties, and can provide controlled drug release profiles. This makes them suitable for sustained drug delivery. In cancer treatment, they are used for selective targeting, enhanced circulation, and controlled release of chemotherapeutic agents. However, One of the main challenges with polymers is achieving the right balance between stability and biodegradability [60]. Additionally, the synthesis and scaling up of polymeric micro/nanoparticles can be complex and costly [30].

Hydrogels are three-dimensional networks of hydrophilic polymers that can absorb and retain large amounts of water, creating soft, tissue-like structures [31]. Hydrogels have high water content, are biocompatible, and can respond to environmental stimuli such as pH and temperature. They are used in drug delivery systems, and as matrices for cell encapsulation [61]. Hydrogels can be designed for controlled and sustained release of therapeutic agents, enhancing the efficacy of cancer treatment. Additionality, they can mimic the properties of cytoplasm. The main challenge with hydrogels is their mechanical strength. Ensuring that they maintain their structure and functionality in the dynamic environment of the human body is crucial [32].

Cell-based materials involve using natural cell components, such as cell membranes or whole cells, to construct artificial cells [62]. This approach leverages the inherent biological functions of natural cells. These materials offer natural targeting capabilities, immune evasion properties, and seamless integration with biological systems. The variability in natural cell membranes can lead to inconsistent results [63]. Additionally, sourcing and processing these materials can be labor-intensive and expensive.

Inorganic materials, such as MOFs and silica nanoparticles, are used to enhance the functionality of artificial cells [64]. These materials are highly stable, have tunable porosity, and can carry large drug loads. Inorganic nanoparticles can be used for imaging, diagnostics, and therapy, providing a comprehensive approach to cancer treatment. Inorganic materials can sometimes be less biocompatible than organic ones, leading to potential toxicity issues [65]. Ensuring their safe and effective use in the body is a key challenge [66].

In hybrid systems, the membrane plays a crucial role in combining the advantages of synthetic and natural materials. For example, using natural cell membranes to coat synthetic cores, creating a biomimetic interface [66]. These membranes provide natural targeting and immune evasion properties, are biocompatible, and can present natural cell surface markers. Similar to cell-based materials, variability and sourcing issues can pose challenges. Additionally, integrating these membranes with synthetic cores requires precise engineering [62].

In conclusion, the choice of materials and approach in building artificial cells for cancer therapy is crucial and depends on the specific therapeutic goals. Each material and method comes with its own set of advantages and challenges, and often, a hybrid approach can provide the most comprehensive solution as shown in Table 2. By leveraging the strengths of different materials and techniques, researchers can develop more effective and personalized cancer treatments [57].

**Table 2:** Materials used for ACs applications, advantages and disadvantages.

Material	Advantages	Disadvantages
**Phospholipids**	They mimic structure and functional of natural cell membranes, facilitating interactions with biological systems [67].	Phospholipid membranes can be less stable and more prone to degradation compared to synthetic materials and Limited functionalization
**Polymer**	They can be easily modified to incorporate various functional groups, enhancing their versatility [68] high targeting and bioavailability, customizable surface properties [69].	The synthesis and assembly of polymer-based can be more complex and costly [28,29].
**Protein**	Proteins can replicate specific functions of natural cells, such as enzymatic activity and signaling	Proteins can be sensitive to environmental conditions and may denature or degrade over time. The production and purification of proteins can be complex and costly.
**Cell-Membrane Derivate**	These materials can help evade the immune system, enhancing their stability and circulation time, better mimic of natural function.	The extraction and incorporation of these materials into ACs can be complex
**Inorganic**	The stability and functionalizability of inorganic materials make it suitable for creating microreactors, where biochemical reactions can be carried out efficiently [70] and High drug loading capacity, pH-sensitive drug release, and tunable porosity [71]	The body may have difficulty clearing inorganic particles, which could lead to accumulation and potential toxicity

### 2.1 Top-down approaches

The top-down approach and the conventional approach has been used to sequentially delete superfluous genes in a natural cell or replace the original genome entirely with a synthetic genome, while still performing specific biological functions [72]. For example, Sun et al. described the development of lymphocyte-based homologous targeting artificial antigen-presenting cells (LC-aAPCs) for personalized cancer immunotherapy [40]. The construction of LC-aAPCs involves using lymphocytes harvested from peripheral blood and employing lipid-DNA-mediated noninvasive live cell surface engineering to achieve the aggregated distribution of peptide-major histocompatibility complex 1 (pMHC-1) and costimulatory ligands on their surface. This allows for the optimized activation of T cells. Lim et al. developed a genetic approach to create cells (SimCells and mini-SimCells) for colorectal cancer therapy [46]. The main difference between SimCells and mini-SimCells lies in their size and genetic composition. SimCells are chromosome-free bacterial cells controlled by designed gene circuits, while mini-SimCells are minicells containing designed gene circuits. Mini-SimCells are smaller in size, typically ranging from 100 to 400 nm, allowing them to penetrate deeply into tumors. In contrast, SimCells are non-replicating and highly controllable, generated from various bacterial chassis such as *Escherichia coli, Pseudomonas putida, and Ralstonia eutropha.* SimCells and mini-SimCells have been engineered to display nanobodies on their surface for specific binding to the carcinoembryonic antigen (CEA) found in colorectal cancer cells. Additionally, mini-SimCells have been engineered to carry cargo gene circuits to produce anticancer compounds, such as converting salicylate into catechol, further enhancing their anticancer properties. These differences in size and genetic composition make SimCells and mini-SimCells suitable for specific applications in targeted cancer therapy. The generation of SimCells is started by the I-CeuI endonuclease-mediated recognition of a specific 26-base pair sequence within bacterial genomes, leading to RecBCD-initiated double-strand degradation. Induction plasmids, exemplified by pRH121, induce SimCells production, offering superior performance over alternative systems. Reprogramming SimCells for cancer cell targeting involves plasmid transformation into E. Coli BL21(DE3) and induction to achieve a highly pure, non-dividing SimCells culture. Furthermore, engineering and purifying mini-SimCells for colorectal cancer cell targeting involve gene knockout to generate chromosome-free mini-SimCells, followed by plasmid transformation for nanobody surface display. Successful mini-SimCells generation and purification entail sequential centrifugation and antibiotic treatment to eliminate parental cells, resulting in highly purified mini-SimCells tailored for targeted cancer [46].

However, there are drawbacks to the top-down approach, especially when it comes to poorly known fundamental mechanisms and components or when there are significant discrepancies in understanding how the system works [13,35]. In these situations, they might need to supplement top-down observations with bottom-up methodologies [73], which involve constructing systems using well-known parts and concepts from the ground up [74]. Integrating functionalized synthetic membranes with biological systems produces "hybrid" ACs. For example, in a study by Zhang et al., the A-NK cells were engineered by decorating natural killer (NK) cells with hepatocellular carcinoma (HCC) specific targeting aptamer (TLS11a) using a metabolic glycan biosynthesis strategy. This approach endowed the NK cells with active targeting ability, allowing them to eliminate any residual unkilled specifically and effectively or resistant tumor cells after photothermal therapy (PTT), thereby enhancing the completeness of tumor removal. The A-NK cells were activated and labeled with Cy5-DBCO via a metabolic glycan biosynthesis, and their active targeting ability was evaluated *in vivo*. The results demonstrated that the A-NK cells, decorated with the HCC-specific targeting aptamer, exhibited excellent active targeting ability, effectively accumulating in tumors and significantly enhancing the antitumor effect [47].

At the nanoscale, natural products of cells, such as EVs, are being explored for cancer treatment. [75,76]. EVs are a natural small cargo-bearing vesicles secreted by cells into the extracellular matrix. Until recently, EVs have been widely explored both as inherent therapeutics in regenerative medicine and as drug delivery systems targeting multiple diseases. Engineering EVs can lead to surface modification, cargo loading and chimeric or hybridization of EVs. These engineered EVs could potentially act as ACs, performing tasks such as targeted drug delivery, tissue regeneration, and even diagnostic applications in cancer field. The low immunogenicity and natural cell-targeting capabilities of EVs make them promising candidates for such application. However, engineering EVs for cancer applications is broadly discuss in literature [[77], [78], [79], [80], [81]].

An emerging focus of interest lies in the use of cell membrane-derived vehicles. This approach incorporates natural cell membranes into artificial systems and is extensively addressed in contemporary literature. This is explored further in Section 2.3 due to its widespread application in these studies, as shown in Table 1.

### 2.2 Bottom-up approaches

**Fig. 4:**
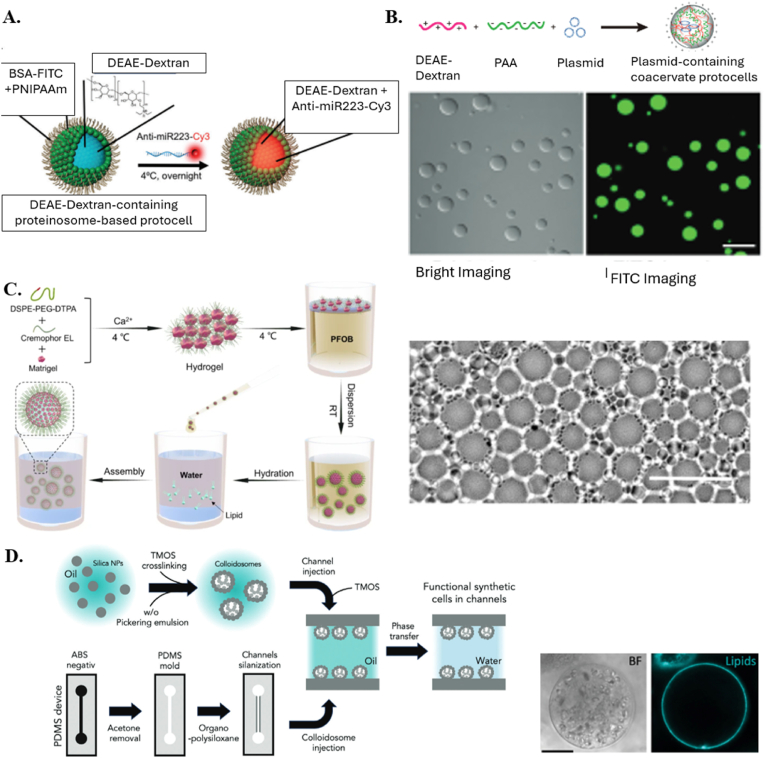
**A.** Schematic representation of dextran-containing FITC-labeled proteinosome-based protocells. Single-channel confocal image of FITC-protocel. Reproduced with permission from Ref. [41]. Copyright © 2024 Wiley-VCH GmbH. **B**. Bright and fluorescence images of the coacervate microdroplets doped with FITC labeled DEAE-dextran. Scale bar: 10.0 μm Reproduced with permission from Ref. [44]. Copyright © 2024 American Chemical Society. **C.** Microscopic photograph of hydrogel dispersing in PFOB. scale bar 100 μm. Reproduced with permission from Ref. [33]. Copyright © 2024 Springer Nature. **D.** Schematic graph of colloidosome ACs inside the microfluidic chip. Representative bright field and confocal microscopy images of the lipid layer (LissRhod B-labeled lipids) formed around silica colloidosomes. The scale bar is 20 μm. Reproduced with permission from Ref. [89]. Copyright © 2024 Wiley-VCH GmbH.

Bottom-up biology is an expanding research field that aims to understand the mechanisms underlying biological processes via *in vitro* assembly of their essential components in synthetic cells [82]. Polymeric ACs are attractive candidates for developing a variety of functional ACs, as presented in Table 1. For example, proteinosomes formed by self-assembling bovine serum albumin/poly(N-isopropylacrylamide) (BSA-NH_2_/PNIPAAm) conjugate enhances the stability and circulation times of ACs. The fabrication involves the synthesis and conjugation of BSA-NH_2_ with PNIPAAm using an emulsion technique involving 2-ethyl-1-hexanol and sonication (Fig. 4A) [41]. Emulsions are mixtures of two immiscible liquids, such as oil and water, where one liquid is dispersed in the other in the form of droplets. Traditional emulsification methods include high-shear mixing, sonication, and high-pressure homogenization. The advantages of emulsions include simplicity, as they are easy to implement with basic equipment, and scalability, making them suitable for large-scale production. However, they have limitations such as limited precision in controlling droplet size and distribution, potential instability due to coalescence and phase separation over time, and high energy consumption [83].

Zhang et al. used small interfering RNA (siRNA) complexes with a low molecular weight polymer poly(β-amino ester) (PBAE) to form stable complexes [84]. The study screened various weight ratios of PBAE to siPlk1, ranging from 1 to 80. The loading efficiency of PBAE was approximately 95 % with PBAE: siPLK1 ratios over 40 and 99 % at the ratio of 50. Another approach to encapsulate nuclear material was developed using electrostatic interaction-mediated liquid-liquid phase separation to create coacervate microdroplets, which have been shown to have high sequestration capability and liquid-like fluidity [44]. The fabrication method of the microdroplets involves the preparation of DEAE-dextran and PAA solutions separately. The coacervates were formed by mixing diethylaminoethyl-dextran hydrochloride/polyacrylic acid (DEAE-dextran/PAA)-based coacervate microdropletin a weight ratio of 0.25 (DEAE-dextran, w/w). The plasmid was sequestered into the coacervate droplets by adding the plasmid to the PAA solution before adding DEAE-dextran, leading to plasmid-containing coacervate microdroplets (Fig. 4B) [44].

Recent advances have led to the development of hydrogel-based ACs that can respond to specific stimuli, such as enzymes, mechanical force, and metabolites [85]. Sodium Alginate (SA) 1.5 % crosslinked by Ca^2+^ ions released from Calcium-Ethylenediaminetetraacetic Acid (Ca–EDTA) upon contact with hydrofluoroether (HFE)-7500 containing 0.15 % acetic acid in a microfluidic device, finding application in the creation of artificial tumor spheroids encompassing diverse cell types and sizes due to its capability of cells to adhere to the alginate [43]. There are other methods to produce ACs by self-assembly techniques to allow the components to come together and form the AC [86,87]. Colloidosomes are vesicles stabilized by colloidal particles. They can encapsulate and release substances in a controlled manner. For example, Matrigel and 1,2-Distearoyl-sn-glycerol-3-phosphoethanolamine-poly(ethylene glycol)-Diethylenetriamine penlaacetic acid (DSPE-PEG-DTPA) were mixed [33]. The combined hydrogel was then chelated at 4 °C with calcium ions, and it was then quickly placed into perfluorooctyl bromide (PFOB). The hydrogel was then shaken at room temperature to solidify it. After that, the hydrogel was spread throughout an aqueous solution that contained lipid molecules. The lipid was finally wrapped around the hydrogel to produce protocells (Fig. 4C). Matrigel, known for its transition from a liquid to a gel state, has proven utility in simulating cell transcription, delivering mRNAs, and reassembling cancer cells within ACs [88]. Despite its versatility, concerns about batch-to-batch variability and potential immunogenicity warrant attention [33].

Hybrid systems put together several advantages and overcome some limitations. For example, Wang et al. created a polymer–lipid membrane nanocarrier through an emulsion method [50]. The process includes dissolving polylactide and hydrogenated soybean phosphatidylcholine in acetone and ethanol, followed by adding the resulting organic phase into a solution of poly-[haemoglobin-tyrosinase](PH-TYR) under moderate magnetic stirring. This forms an emulsion, which is then stirred continually for 30 min. The resulting organic solvent is removed using a rotary evaporator under vacuum for 1 h. The nanocapsules are then separated by centrifugation and concentrated with filtration.

In a recent study, Hakami et al. introduced a novel approach to integrating synthetic cells into 3D microfluidic devices, specifically designed for organ-on-chip technologies [89]. The authors utilized silica colloidosomes anchored to PDMS microfluidic channels via tetramethyl orthosilicate (TMOS)-based cross-linking, forming dense networks. Silica colloidossomes produced through emulsion with a mixture of 2 % sodium alginate and bovine serum albumin (BSA) is prepared in a 1:1 ratio. This mixture served as the aqueous phase for the formation of colloidosomes containing the hydrogel core. Then Small unilamellar vesicles (SUVs) were prepared by extrusion, mixing individual lipids dissolved in chloroform in the desired ratio and drying them in a vacuum chamber. The lipid film was resuspended in PBS and extruded through a 100 nm diameter pore filter. SUVs were added to the colloidosomes to form a lipid layer on the colloidosome surface as displayed in Fig. 4D.

As an overview of the bottom-up techniques used to create ACs for cancer treatment, Table 3 presents the advantages and disadvantages of the fabrication processes discussed above. Self-assembly and phase separation are the driving mechanisms for many of these methods, including solvent evaporation, emulsion polymerization and *in situ*/interfacial polymerization, salting-out, and phase inversion [90]. Microfluidic and capillary-based approaches can generate highly monodisperse populations of microparticles, although these methods are inherently less high-throughput due to their setup [91].

**Table 3:** Advantages and Disadvantages of the fabrication techniques used to create ACs.

Technique	Advantages	Disadvantages
**Emulsion** **Micro emulsion**	Simple, cost-effective, high encapsulation efficiency	Limited control over size, stability issues
**Microfluidics**	High precision, reproducibility, complex structures [92]	Requires specialized equipment, costly
**Self-Assembly**	Mimics natural processes, highly ordered structures	Limited control, sensitive to environment
**Thin Film Hydration**	Simple, high encapsulation efficiency	Heterogeneous sizes, requires additional steps for uniformity
**Double Emulsion**	High encapsulation efficiency, multi-compartment structures	Complex, stability issues
**Coprecipitation**	Simple, cost-effective, uniform particles	Limited control over size and composition, potential impurities

These techniques are being actively researched and refined to improve the delivery and efficacy of cancer treatments. By leveraging the precision, control, and biocompatibility offered by bottom-up approaches, researchers can develop more effective and targeted cancer therapies. Additionally, the full molecular composition of synthetic cells is know since they are constructed from scratch which is not the case with living cells. In general, bottom-up approaches are far less complex than conventional cell therapies and can be programmed to do specified functions, allowing complete control and predictability over the variables. Lastly, they raise fewer concerns about safety and ethical aspects [93,94].

Given the complexity and variability of cancer therapy, a hybrid approach is often the most advantageous. It allows for the precise control and utilization of natural cellular functions while incorporating innovative synthetic components to enhance targeting, delivery, efficacy and other applications as phototherapy or diagnostic. This approach can provide a comprehensive solution that addresses the limitations of both top-down and bottom-up methods, offering the best potential for effective and personalized cancer treatments.

### 2.3 Encapsulation of ACs

Table 1 diversity reflects the complexity of replicating natural cellular functions as well as the diversity of the materials and fabrication approaches that can be explored. Additionally, the presented systems explored different functions. Functional modules in ACs include compartmentalization, which creates distinct areas within the cell to separate different biochemical processes, similar to organelles in natural cells [17,95]. Energy supply modules generate or store energy, such as synthetic systems that produce ATP using light or other sources. Metabolism modules consist of enzymatic networks that facilitate metabolic reactions, allowing the artificial cell to process nutrients and produce necessary compounds [96]. Gene replication and expression systems enable the replication of genetic material and the synthesis of proteins, mimicking the central dogma of molecular biology [97]. Some of these functions were not yet apply in Cancer Therapy.

**Fig. 5:**
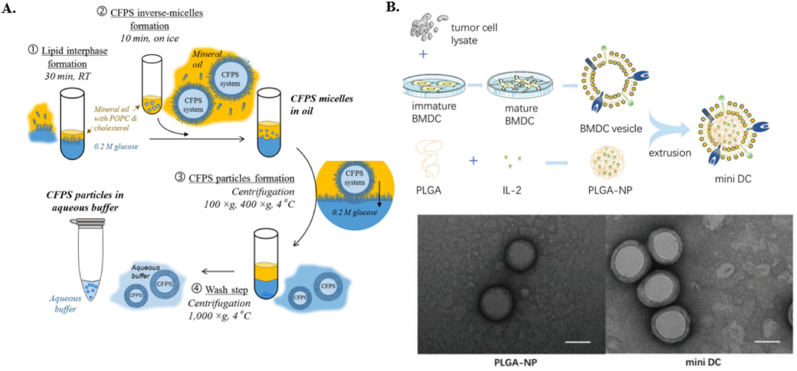
**A.** The schematic diagram of the preparation of the Liposomes. Reproduced with permission from Ref. [102]. Copyright © 2024 Wiley-VCH GmbH. **B**. Schematic illustration for the preparation of mini-DC, which were fabricated by coating IL-2 loaded PLGA-NP with tumor cell lysate-primed DC membrane. Representative TEM images of PLGA-NP (left) and mini-DC (right) stained with uranyl acetate. Scale bar: 100 nm. Reproduced with permission from Ref. [105]. Copyright © 2024 Wiley-VCH GmbH.

In general, lipid-based ACs can encapsulate both hydrophilic and hydrophobic cargo [39]. However, Lipid ACs often have poor retention efficiency due to their high permeability, influenced by their chemical properties, size, saturation levels, and preparation methods [98,99]. The permeability of lipid vesicles may pose challenges in controlling the exchange of molecules between the cyst and its surroundings, affecting the functionality. Lipid vehicles may have limited stability, especially in complex environments or over extended periods [100,101]. Encapsulating cell-free systems for protein synthesis is one of the functions of ACs. This approach leverages the ability of ACs to create a controlled environment that mimics the intracellular conditions necessary for protein synthesis. POPC (1-palmitoyl-2-oleoyl-sn-glycero-3-phosphocholine) and cholesterol (1:2 M ratio) were used to prepare liposomes encapsulating cell-free protein synthesis (CFPS) through emulsion formation, as displayed in Fig. 5A [102]. The study successfully demonstrated the synthesis of Pseudomonas exotoxin A (PE) inside the vesicles, leading to significant cell death in 4T1 breast cancer cells in culture concluding that the synthetic lipid-based vesicles can be used as effective platforms for synthesizing therapeutic proteins within tumors [102].

Single or multiple emulsions are complex systems where emulsions are nested within each other. They are stabilized by surfactants, which reduce the interfacial tension between the two liquids [103,104]. The most common types are water-in-oil-in-water (W/O/W) and oil-in-water-in-oil (O/W/O). For example, the fabrication method of PLGA-NP involves a double-emulsion technique. Initially, an aqueous solution containing the desired therapeutic agent, such as interleukin-2 (IL-2), was added to a solution of PLGA polymer in a water-immiscible organic solvent, typically dichloromethane. This mixture was then emulsified by sonication and added dropwise to a continuously vortexed tube containing a stabilizer, such as polyvinyl alcohol (PVA). The resulting double emulsion was further sonicated and then transferred to a vacuum aspirator to remove the organic solvent as shown in Fig. 5B [105]. The emulsion methods allow for adjusting lipid composition and protein/cargo concentrations to achieve the desired size distribution, internal microstructure, and mechanical properties. However, generally, this method produces less monodisperse solutions of ACs. Achieving a controlled release of encapsulated compounds can be difficult, as it depends on the stability of the emulsion and the interaction between the encapsulated compounds and the emulsion matrix.

Inorganic NPs offer unique properties for designing biomimetic ACs. Researchers have successfully loaded them with anti-tumor drugs, transforming them into miniature drug carriers that mimic the function of granzymes, enzymes naturally produced by immune cells to kill cancer cells [52]. Barium sulfate (BaSO_4_) NPs engineered to mimic the functions of macrophages. Hierarchical nanostructure of this system allows to efficiently capture tumor antigens, molecules that trigger an immune response. Once captured, these antigens are presented to T cells, stimulating a targeted attack on cancer cells [54]. The BaSO_4_@Zeolitic imidazolate framework-8/transferrin (BaSO_4_@ZIF-8/TRF) NM was synthesized through a series of chemical reactions, including the preparation of BaSO_4_ NPs, the growth of ZIF-8 shells, and the functionalization with transferrin (TRF) for tumor targeting. Similar as proinflammatory cytokines, Zn^2+^ can trigger cell anoikis to expose tumor antigens, which are selectively captured by the BaSO_4_ cavities. Therefore, the hierarchical nanostructure allows them to mediate immunogenic death of tumor cells and subsequent antigen capture for T cell activation to enhance long-term antitumor immunity. The immune response and antitumor effects were assessed in 471 tumor cells and J774A.1 and BALB/c mice [54].

In another study, Rahikkala et al. created two types of hybrid NPs [45]. The first type involves the encapsulation of porous silicon NPs (UnPSi NPs) within red blood cell (RBC) membranes, functionalized with a cell membrane-mimicking block copolymer polydimethylsiloxane-poly(2-methyl-2-oxazoline)- tyrosinase-related protein 2 (PDMS-PMOXA-TRP2). The second type consisted of UnPSi NPs surface-functionalized with TRP2 peptides, which are then encapsulated within RBC membranes. When in the presence of peripheral blood immune cells (PBMCs), these NPs can induce apoptotic cell death of T cells, with the encapsulated antigens leading to a stronger T cell deletion [45]. Tramontano et al. described a co-flow microfluidic used to encapsulate the DNPs in a gastro-resistant polymer, allowing for a sustained release of galunisertib at the intestinal pH [56]. The advantages of using this device include precise encapsulation of the NPs and high batch-to-batch reproducibility. In general, monodispersity is the highest in microfluidic methods and they are the only ones offering high-throughput production of same-sized ACs. The possibility to control the shape and size of ACs is the major advantage of microfluidics [82]. However, their larger size (around 400 nm) poses a limitation. This size makes them unsuitable for intravenous injection, restricting their administration route to oral ingestion [56].

Encapsulation of drugs inside ACs can be achieved using various methods, such as microencapsulation, nanoencapsulation, and spray drying [106]. Distinct types of intercellular agents can be incorporated. Small molecule chemotherapeutic agents, such as doxorubicin and Galunisertib, are commonly encapsulated within ACs, as shown in Table 1. Macromolecular drugs, such as monoclonal antibodies, recombinant proteins, peptides (melittin), and nucleic acids (*e.g.*, siRNA and mRNA) can also be encapsulated within ACs. These biological therapeutics target specific signalling pathways or molecular targets implicated in cancer progression, offering targeted and personalized treatment options. Xu et al. developed non-interfacial self-assembly (NISA) nanodroplets to create ACs as non-viral mRNA vehicles for transfecting 293T cells. Encapsulation of DNA templates and RNA transcriptase within these ACs allowed for effective mRNA transcription and protection, although the yield was lower compared to *in vitro* kits. The ACs facilitated efficient mRNA protection and interaction with 293T cells [33].

Indocyanine green (ICG) is a near-infrared fluorescent dye commonly used for imaging-guided cancer therapy and photodynamic therapy (PDT). As an imaging agent, ICG enables real-time visualization of tumor tissues and helps image-guided surgical procedures. Moreover, ICG can also function as a photosensitizer in PDT, like chlorin e6, by generating cytotoxic ROS upon light activation. Encapsulation of ICG and chlorin 6 within ACs enhances its stability, bioavailability, and tumor-targeting capabilities for combined imaging and therapeutic applications. Wang et al. improved the biorthogonality of zwitterionic choline phosphate via a ring-opening reaction to enhance the DOX loading efficiency and cellular uptake, aiming to improve breast cancer cell cytotoxicity. Folate-conjugated choline phosphate, synthesized via *in situ* azide-alkyne click reaction, was loaded with DOX and showed controlled release and enhanced cellular uptake, resulting in higher treatment efficacy in 4T1 tumor-bearing mice [107].

**Fig. 6:**
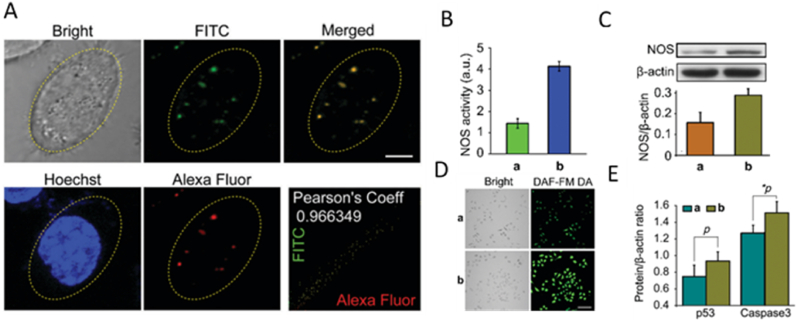
Plasmid transfection through coacervate microdroplets. **A.** Fluorescence imaging assay after 2.5 h incubation to observe coacervate protocells cellular internalization. Green and red fluorescence indicate coacervate protocells and blue indicates living cells nuclei. **B.** Relative NOS activity in transfected cells by free and entrapped pNOS. **C.** NOS expression in transfected cells normalized against the internal control (∗p = 0.04). **D.** Confocal fluorescence images of transfected cells by (a) free and (b) pNOS-coacervates. Scale bar = 30 μm (E) Protein levels of p53 and Caspase-3 in transfected cells by (a) free and (b) pNOS-coacervates (*p* = 0.13, ∗*p* = 0.04). Reproduced with permission from Ref. [44]. Copyright © 2024 The Royal Society of Chemistry. (For interpretation of the references to color in this figure legend, the reader is referred to the Web version of this article.)

Other types of ACs, such as liquid coacervates, have also been investigated for cargo delivery. A recent study conducted by Zhang et al. showed the advantage of liquid coacervate microdroplets as membrane-free compartment protocells with their unique liquid-like fluidity and sequestration capability properties. In this study, oppositely charged diethylaminomethyl-dextran/polyacrylic acids were associated via the liquid-liquid phase separation. Nitric oxide species (NOS) plasmid was sequestrated within the coacervate microdroplets for efficient gene transfection and nitric oxide (NO) endogenous overproduction, a recognized therapeutic agent capable of inducing cell apoptosis via mitochondrial respiration blocking, which subsequently causes reversible inhibition of electron transfer, for tumor cell therapy. Plasmid encoding enhanced green fluorescent protein (pEGFP) was successfully loaded in the coacervates at 12.5 mg/mg, which confirmed the high sequestration capability of coacervates (about 99.8 %). The structure of plasmid-loaded coacervates after internalization into cells remained intact (Fig. 6A). Furthermore, the pH-sensitive coacervates were able to dissociate in lysosomes and facilitate the plasmid assimilation, as revealed by propidium iodide (PI) fluorescence staining. The increased NOS activity of living cell, transfected with pNOS-loaded coacervates, compared to cells transfected with free pNOS was confirmed by enhanced enzymatic activity (2.5-fold) and NOS protein levels quantified by western blot (1.8-fold), intense and uniform green fluorescence, because of l-arginine conversion into NO (Fig. 6B–D). The authors also verified the efficient intracellular NO production (up to 12.6 μm) and the induction of apoptosis by increased levels of apoptosis-associated proteins such as p53 and Caspase-3 (Fig. 6E) [44].

A novel study developed a synthetic cell that encapsulate the PURExpress [108]. The PURExpress In Vitro Protein Synthesis Kit was used for CFPE. This system contains all the necessary components for transcription and translation, including *E. coli* protein expression machinery. The study successfully demonstrates that RNA thermometers (RNATs) can regulate protein expression within synthetic cells based on temperature. The synthetic cells were engineered to synthesize the membrane pore protein α-hemolysin (αHL) *in situ*. This protein self-inserts into the synthetic cell membrane and facilitates the release of pre-encapsulated small-molecule cargo (calcein). This study paves the way for innovative approaches to cancer treatment, where precise control over drug delivery can lead to better outcomes for patients.

Moreover, encapsulating anti-cancer drugs within ACs protects against enzymatic degradation and immune clearance, prolonging the circulation time of the drugs in the bloodstream and improving their bioavailability. This enhanced stability and pharmacokinetic profile. Integrating stimuli-responsive materials makes fabricating ACs capable of efficiently reacting to external triggers possible. This innovation bears significant promise for developing controllable and adaptable micro-reaction systems.

Maintaining the stability and functionality of encapsulated cell-free systems over time is difficult. These systems can degrade or lose activity, especially in complex biological environments. Achieving high encapsulation efficiency while maintaining the functionality of the encapsulated components is difficult. Inefficient encapsulation can lead to loss of valuable materials and reduced system performance. Finally, achieving standardization and reproducibility in the design of ACs is challenging due to the variability in materials and methods.

### 2.4 Surface engineering of ACs

**Fig. 7:**
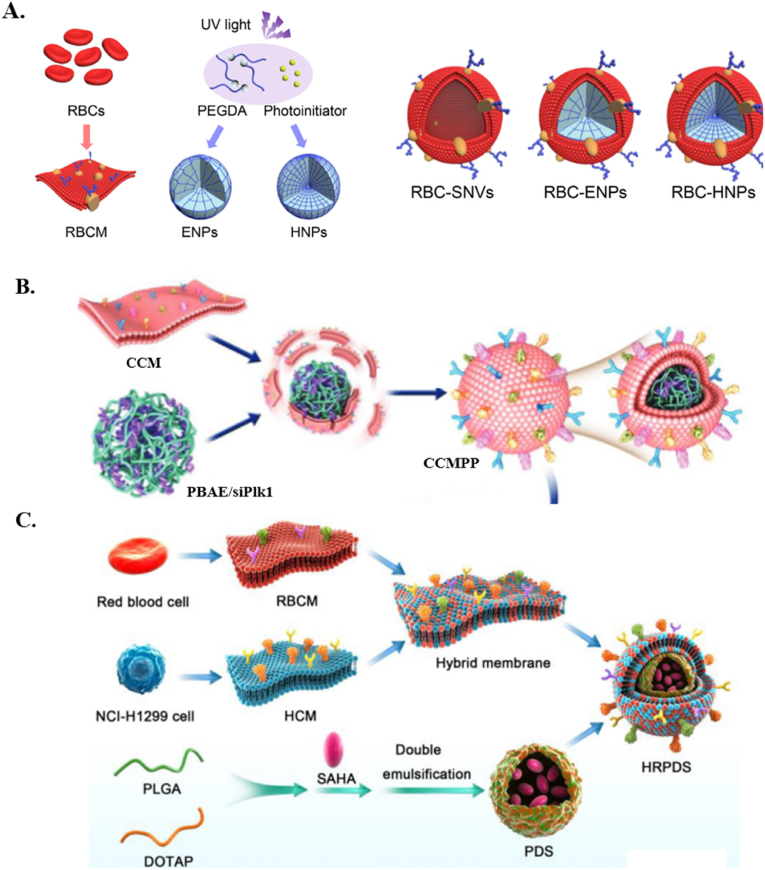
**A.** Bionic design of RBC-derived nanocarriers. Reproduced with permission from Ref. [51]. Copyright © 2024. The Royal Society of Chemistry. **B.** The schematic diagram of the preparation of the cell cancer membrane particle and the cellular uptake. Reproduced with permission from Ref. [84]. Copyright © 2024 Wiley-VCH GmbH. **C.** Scheme illustration of biomimetic nano vehicle-mediated epigenetic inhibition in metastatic lung cancer. Reproduced with permission from Ref. [49]. Copyright © 2024 Elsevier B.V.

Cell membranes separate the cell from the extracellular environment and have a key role in physiological processes, such as signalling, metabolism, and communication [109]. Engineering the surface of ACS is important due to its role in creating high-functional bio-interfaces [110]. Cell mimics encapsulated by lipid membranes are a powerful tool to reconstitute the biological machinery of living cells [111]. In the studies presented in Tables 1 and it is possible to distinguish two different strategies for engineering ACs, as also shown in Fig. 7. The first is using natural cell membranes to trigger or escape the immune system [112]. The second is to build the membrane surface using lipids, polymers, proteins, and peptides [93].

The approach to membrane isolation can vary depending on the specific cell type. For example, it is easier to extract membranes from anucleate cells like RBCs than eukaryotic cells [113]. ACs using natural membranes show some advantages of immune escape ability and achieve long-term circulation in the blood due to the same surface components from natural cell membranes as RBC and (Bone Marrow-Derived Cells) BMDCs [114].

RBC membranes have several unique characteristics that make them ideal for use, including the absence of organelles, their ability to form double concave cells, their ability to be safely isolated from the body, and to serve as an abundant membrane for coating [115]. These RBCs may easily circulate in the bloodstream for up to four months, which is why NPs covered with RBC membranes have a longer circulation duration than NPs without the membrane [116]. Additionally, NPs are biodegradable and pose no threat to biological systems. Intrinsic membrane surface molecules from the RBC membrane cause NPs to evade the immune system. To replicate disabled, dynamic, and aged RBCs, an artificial nanoscale RBC-mimetic system was built using PEGDA hydrogel NPs and RBC membranes (Fig. 7A). It was anticipated that RBC membrane-coated elastic PEGDA NPs (RBC-ENPs), which mimicked native dynamic RBCs, would have an enhanced capacity to elude the immune system and traverse the constricted extracellular space of tumors [51].

Cancer cell membranes (CCM) may be used in both directions to enhance anticancer therapies and to effectively transfer tumor-associated antigens to APCs and trigger an anticancer immune response or immunological adjuvants bind to the antigens. Furthermore, they exhibit a homotypic binding mechanism in tumor source cell-specific targeting, which is frequently observed in tumor cells, and they retain the same cell adhesion molecules as the original tumor source cells [117]. Cancer membrane-camouflaged nanocarriers, which functionalize nanocarriers with tumor-targeting capacity to homologous cancer cells, can also boost the efficacy of targeted administration [118]. However, using natural components like cancer cell membranes may raise ethical concerns. Fig. 7B shows one example here H1299 cells were subjected to a freeze-thaw process combined with sonication and extrusion to prepare CCM-coated ACs. The freeze-thaw process involves freezing the cells at extremely low temperatures and thawing them either at 25 °C or 37 °C. Repeating this process results in cell lysis and the membrane can be isolated through centrifugation or other methods [119]. CCM facilitates homotypic targeting, but technical challenges like the negatively charged surface of CCM, which lowers the encapsulation efficiency of similarly charged therapeutic nucleic acids, limit its widespread application. One successful example utilized CCM to enhance the stability and gene-silencing effect of PBAE/siRNA NPs. The surface antigenic diversity replicated by CCM led to higher cellular uptake. siRNA bound to PBAE at a weight ratio over 1:50 ensured high loading efficiency (95 %) and protection from enzymatic degradation, resulting in significant downregulation of Plk1 mRNA and induced apoptosis in cancer cells. PBAE's pH-responsive structure facilitated siRNA release in acidic environments like endo/lysosomes, enabling effective RNA silencing and apoptosis in cancer cells. The ACs demonstrated specificity in targeting cancer cells while minimizing uptake by normal cells, such as rat myocardial and human umbilical cord-derived mesenchymal stem cells, and macrophages. *In vivo* studies in NCl-H1299 tumor-bearing mice showed significant tumor reduction in AC-treated groups compared to controls [84]. Another study utilized a hybrid cell membrane to camouflage loaded poly(lactic-co-glycolic acid) (PLAG) and 1,2-dioleoyloxy-3-(trimethylammonium) propane (DOTAP) NPs (PDS) loaded with suberoylanilide hydroxamic acid (SAHA) to inhibit lung cancer liver metastasis. Encapsulating SAHA in pH-sensitive NPs made of PLGA and DOTAP, and coating them with hybrid membranes from RBCs and NCL-H1299 cells, reduced non-specific uptake and improved lysosomal escape. Treated NCI-H1299 cells showed effective tumor inhibition and apoptosis, attributed to enhanced expression of apoptosis-inducing genes. *In vivo* these NPs demonstrated prolonged circulation and effective liver targeting in BALB/c mice, contributing to higher antimetastatic efficacy (Fig. 7C) [49].

Surface engineering is widely utilized to effectively target cells as it is crucial for effective drug delivery. Targeting moieties are covalently conjugated and coated on the surface of ACs to improve their selectivity and affinity to specific cells, such as cancer cells. Notably, materials like polyethylene glycol (PEG), widely used in membrane technologies can improve circulation time and reduce immune response to ACs.) [52,120].

In the context of the lymph-node-on-a-chip model, ligands like anti-CD3 and anti-CD28 antibodies are used to engineer the surface of ACs [89]. These ligands bind to specific receptors on the T cell surface, initiating signaling pathways that lead to T cell activation and proliferation. They are crucial for mimicking the activation signals provided by antigen-presenting cells in natural immune responses.

More sophisticated systems were design as the study conducted by Soogard et al. where an artificial receptor capable of performing transmembrane signalling in synthetic cells was presented [121]. This involved designing a receptor that could be activated by an external enzyme and release a secondary messenger into the cell. The artificial receptor is specifically designed to be activated by the enzyme β-glucuronidase (GUS). When GUS is added to the extracellular environment, it binds to the receptor and triggers a chemical reaction. As the self-immolate linker decomposes, it releases L-cysteine, a thiol-containing amino acid. L-cysteine acts as the secondary messenger in this system and diffuse across the lipid bilayer. Inside the cell, L-cysteine acts as a reducing agent. It reacts with a disulfide-protected cysteine protease, which is in an inactive zymogen form. The reaction between L-cysteine and the disulfide bond of the proteose leads to its activation. The development of the artificial receptor represents a significant advancement in synthetic biology. It allows for the creation of synthetic cells that can mimic the sophisticated signalling and response mechanisms of natural cells.

Another study by Seo et al. explored two types of polymersomes, Pluronic and oly(butadiene)-b-poly(ethylene oxide) (PB-PEO), which were synthesized using droplet microfluidics [122]. These polymersomes exhibited different permeabilities to hydrophilic molecules, allowing selective induction of enzymatic reactions. Their study aimed to demonstrate the programmability of enzymatic reaction networks within polymersomes, achieving precise control over biochemical reactions. For that reason, diverse models of enzymatic reactions such aspyruvate kinase (PyK), hexokinase (HK), glucose-6-phosphate dehydrogenase (G6PDH), and lactic dehydrogenase (LDH), were integrated into the polymersomes. The polymersomes act as both sender and receiver cells. For example, Pluronic polymersomes (sender cells) generate pyruvate through the PyK reaction and release it into the surrounding media. The released pyruvate diffuses into adjacent PB-PEO polymersomes (receiver cells), where it triggers the LDH reaction, consuming NADH and producing NAD^+^. The polymersomes can mimic cell-to-cell communication by selectively permeating substrates and triggering enzymatic reactions in adjacent cells. Polymersomes with selective permeability can be engineered to encapsulate and release drugs in response to specific enzymatic signals, enhancing the targeted delivery of cancer therapeutics.

Generating membrane-based ACs in a controlled way and high throughput is a key obstacle, including the need for precise parameters such as size, biomolecular composition, and spatial organization [123]. This limits their application, necessitating further improvement [124]. To design an artificial membrane achieving selective permeability akin to natural membranes is a key consideration. This selective permeability regulates the flow of molecules into and out of synthetic cells, mimicking the behavior of biological systems [125].

## 3 Applications of ACs

### 3.1 Biomimetic ACs to enhance drug delivery and diagnostics in cancer therapy

By encapsulating chemotherapeutic agents, biomimetic ACs can deliver these drugs directly to cancer cells, reducing systemic toxicity and improving treatment outcomes. In a study by Miao et al., RBC-membrane camouflaged nanocarriers were prepared to mimic both the physical and biological properties of RBCs, and to study how the deformability and stiffness of RBCs affect their biological behavior [51]. The developed RBC membrane-coated poly (ethylene glycol) diacrylate NPs (RBC-ENPs) were utilized to simulate diseased, dynamic, and aged RBCs. They displayed minimum immunoglobulin adsorption in the protein corona surface, high immunocompatibility and ultralong circulation. Furthermore, their deformation properties along with the excellent penetration into multicellular spheroid and tumor accumulation reinforced their utility as a chemotherapeutic DOX-loaded PEGylated liposomes [51].

In another study, Ni et al. constructed an AC carrier by coating silica NPs (SIP) by CCM for chlorin e6 (Ce6) delivery, as a PDT for tumor disruption, while enhancing immune response stimulation through cytokines secretion to promote anticancer efficacy [55]. The hemolysis and protein absorption tests confirmed the biocompatibility and inertness to BSA of CCM-coated SIP, compared to free SIP NPs, which might be due to the negative surface charge. Moreover, the drug release behavior showed much lower release after 120 h incubation in acidic environment (35.15 %) compared to the physiological environment (79.74 %), which indicated the favorable effect of CCM-coated SIL NPs in malignant tumors. Moreover, the anticancer effect of CCM-coated SIL NPs conducted *in vitro* in MCF-7 cancer cells, showed that the CCM-coated NPs could exert benefits with lower cancer cell viability and downregulation of Caspase-3 and Cytochrome *c* compared to other groups, which are indicators of apoptosis initiation. The *in vivo* anticancer study in MCF-7 tumor-bearing mice was also consistent with *in vitro* results, confirming the preferable biocompatibility, the tumor homing, and shielding property, and tumor tissue apoptosis to promote the anticancer efficacy and reducing undesired effects, which were attributed to the CCM-mediated endocytosis and their tumor targetability [55].

**Fig. 8:**
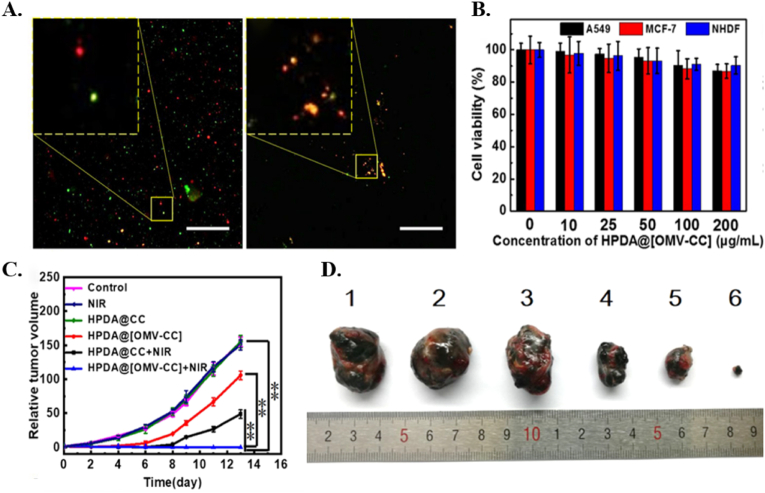
**A.** Distinct red and green fluorescence images of OMVs and CC membrane and colocalization of fluorescence signals in the fused membrane revealed by confocal laser scanning microscopy (CLSM) images. **B.** Cytotoxicity of HPDA@[OMV-CC] NPs showed high biocompatibility (>90 %) when the concentration of NPs was up to 200 μg/mL. **C.** Relative tumor volume of melanoma-bearing mice after receiving different treatments show an obvious antitumor performance in HPDA@[OMV-CC] NPS compared to the control group, neither HPDA@CC NPS nor NIR irradiation alone Data were represented as means ± SD. n = 5 for panels (B) and (C). NS indicates no statistical difference, and ∗∗ indicates *P* < 0.01. **D.** Photographs of tumors explanted from melanoma-bearing mice at 13 days after treatment with (1) PBS, (2) NIR irradiation, (3) HPDA@CC NPs, (4) HPDA@[OMV-CC] NPS, (5) HPDA@CC NPs with NIR irradiation, and (6) HPDA@[OMV-CC] with NIR irradiationReproduced with permission from Ref. [42]. Copyright © 2024 Wiley-VCH GmbH. (For interpretation of the references to color in this figure legend, the reader is referred to the Web version of this article.)

In another relevant study, Wang et al. used a combination of B16-F10 CCM and *E. coli* outer membrane vesicle (OMV) to coat hollow polydopamine (HPDA) NPs and obtain HPDA@[OMV-CC] NPs [42]. The fusion and colocalization of CC and OMV were verified by the exhibition of distinct fluorescence signals, and the retain of the protein characteristics was shown by Sodium dodecyl-sulfate polyacrylamide gel electrophoresis (SDS-PAGE) protein analysis, as depicted in Fig. 8A. *In vitro* experiments showed good biocompatibility (about 95 % cell viability) and homotypic-targeting capability by increased fluorescence intensity of dyed-HPDA@[OMV-CC] NPs in B16-F10 cells than other cell types (NHDF and MCF-7) upon 4 h of incubation (Fig. 8B) [42]. Furthermore, the PTT mediated by HPDA NPs together with the immune therapeutic functions of OMV-CC membrane showed enhanced antitumor multifunctionality towards melanoma *in vivo*. The antitumor immune response triggering effect of OMV was investigated by quantification of interleukin-12 (IL-12p40) and interferon (IFN)-ɣ cytokines in blood serum and tumor. The levels of both cytokines sharply increased during the first hours and then gradually reduced to the normal level, for C57BL/6 mice treated with HPDA@[OMV-CC]. The rate of tumor growth inhibition for mice treated with HPDA@[OMV-CC] and NIR irradiation showed nearly 99.9 % inhibition, which showed that combination of PTT and antitumor immune response exhibited an advanced antitumor performance (Fig. 8C–D).

In PTT-based strategies, heat stress led to overexpression of heat shock proteins 70 (HSP70), which causes tumor chemoresistance. To enhance the PTT efficacy, in a study by Zhang et al., a sequential therapy, photothermal combined with the transfer of artificial natural-killer cells, was adopted for hepatocellular carcinoma. DNAzyme, as a mRNA cleavage, and polyetherimide were absorbed on the surface of Mn^2+^ coordinated tetrahydroxyanthraquinone to act as an anti-heat approach. Natural-killer ACs were decorated with HCC-specific targeting aptamer and their synergistic effects were investigated in HepG2 tumor-bearing mice treated with PTT 2 days before intravenous injection of ACs. *In vitro*, the targetability and cell-killing effect of the engineered ACs was confirmed by the significant difference of viable HepG2 cells when treated with engineered ACs (24.22 %), compared to unengineered ACs (52.26 %). The *in vivo* results showed that although ACs injection and anti-heat endurance PTT alone provided some degrees of tumor growth inhibition, the combinational therapy showed significantly improved tumor growth inhibition [47].

### 3.2 AC as a tool for cancer research

#### 3.2.1 Model for studying drug permeability and mechanisms of chemoresistance

While lipid vesicles are simpler and primarily used for encapsulation and delivery, ACs are more complex systems designed to mimic the structure and function of natural cells, such as mimic the fluidity and symmetry of natural cells. For example, Stephenson et al. described a microfluidic platform with an aqueous solution (buffer with DOX) and the oil phases (different lipid formulations in squalene) meeting at two T-junctions, allowing the formation of two different types of droplets surrounded by different lipid monolayers [39]. ACs were made from naturally derived lipids, including l-a-phosphatidylcholine (PC), l-a-phosphatidylethanolamine (PE), sphingomyelin (SM), and cholesterol (CHOL). These lipids were selected based on lipidomic data that quantify the asymmetry in mammalian cell membranes [39]. Two different types of asymmetric droplet interface bilayers (DIBs) were achieved: 1) symmetric DIBS formed using a 50:50 mix of the asymmetric lipid formulations for both droplets and 2) blend DIBs formed using a 75:25 mix of the asymmetric lipid formulations for the outer leaflet and a 25:75 mix for the inner leaflet. By closely mimicking the asymmetry found in mammalian cells, the ACs offer a more accurate model for studying drug permeability and mechanisms of chemoresistance. Microfluidics in this work enabled stable cell-sized asymmetric DIBs. The advantage of using microfluidics lies in its ability to precisely control the formation of asymmetric bilayers using naturally derived lipids that mimic mammalian cells and high encapsulation efficiency [[126], [127], [128]]. Additionally, the microfluidic platform facilitates the quantification of the effect of lipid asymmetry on drug permeability, specifically focusing on the chemotherapy drug, DOX, revealing that bilayer asymmetry has a measurable impact on membrane permeability to the chemotherapeutic drug.

#### 3.2.2 Biomimetic tumor microenvironment

**Fig. 9:**
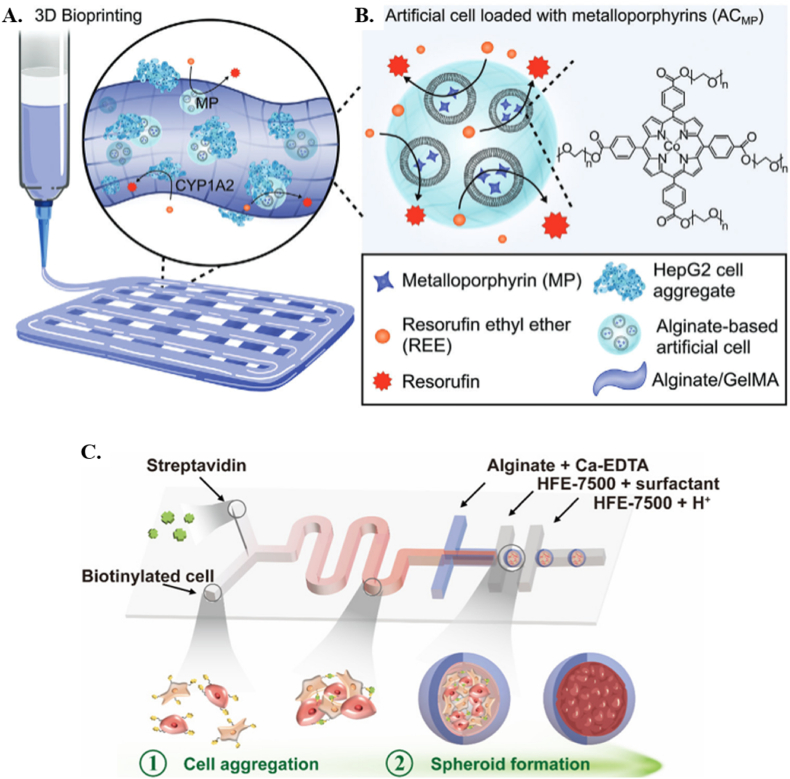
**A.** Semi-synthetic tissue is obtained by 3D printing of a composite bioink consisting of HepG2 cell aggregates and artificial cells as the solid phase and alginate/gelatin methacryloyl (GelMA) as the liquid phase. HepG2 cells express CYP1A2 enzymes that convert resorufin ethyl ether (REE)to resorufin. **B.** alginate-based artificial cells are equipped with metalloporphyrins (MP) that mimic the catalytic activity of CYP1A2. CYP1A2 is anenzyme from the cytochrome P450 family, which is responsible for metabolizing various substances, medications, and environmental toxins, in the liver. Reproduced with permission from Ref. [133]. Copyright © 2024. Wiley-VCH GmbH. **C.** Gradual cell spheroid formation and encapsulation with a microfluidic deviceReproduced with permission from Ref. [43]. Copyright © 2024 American Chemical Society.

Designing and constructing a biomimetic tumor microenvironment via biomimetic cell-based vesicles is another viable application of ACs [129]. Scaffold-free multicellular tumor spheroids formation that mimics the heterogeneous and architectural complexity of a solid cancer is another vital example of biomimetic systems [130]. These self-organized aggregates have a short formation period and because of their metabolic gradient, they can mimic the radiation and medication resistance of human tumors [131]. Westensee discusses the fabrication and integration of ACs with HepG2 cells in 3D-bioprinted structures to enhance liver-like functions as shown in Fig. 9A–B [132]. Artificial cells, engineered to mimic the enzyme activity of the cytochrome P450 family, were incorporated into a composite bioink alongside HepG2 cell aggregates. The resulting semi-synthetic tissue demonstrated boosted CYP1A2 activity over 35 days and the structure maintained its integrity. In a study, Hou et al. developed spheroids using droplet-based microfluidics and cell membrane engineering within an alginate shell (Fig. 9C). Tumor cell lines (HCT116, A549, HepG2) and fibroblast NIH3T3 cells were rapidly aggregated and encapsulated in alginate microspheres, controlled by flow rates and ratios. The alginate shell prevented cell extravasation and spheroid fusion, while biotin and streptavidin enhanced cell adhesion and proliferation. The spheroids formed gradually, showing high cell viability and formation yield (>80 % and >90 %, respectively). The spheroids were released from the alginate shell through reversible ionic crosslinking. The system's applicability was tested by evaluating Vascular endothelial growth factor (VEGF) secretion and 5-fluorouracil (5-FU) resistance. Co-culture spheroids showed increased VEGF secretion and angiogenesis compared to monotypic models, indicating effective cell-cell interactions. Additionally, co-culture spheroids demonstrated higher cell viability and greater 5-FU uptake, suggesting their potential for drug screening and understanding tumor environment-drug resistance connections [43].

### 3.3 ACs and biomimetic immunotherapy modulators

**Fig. 10:**
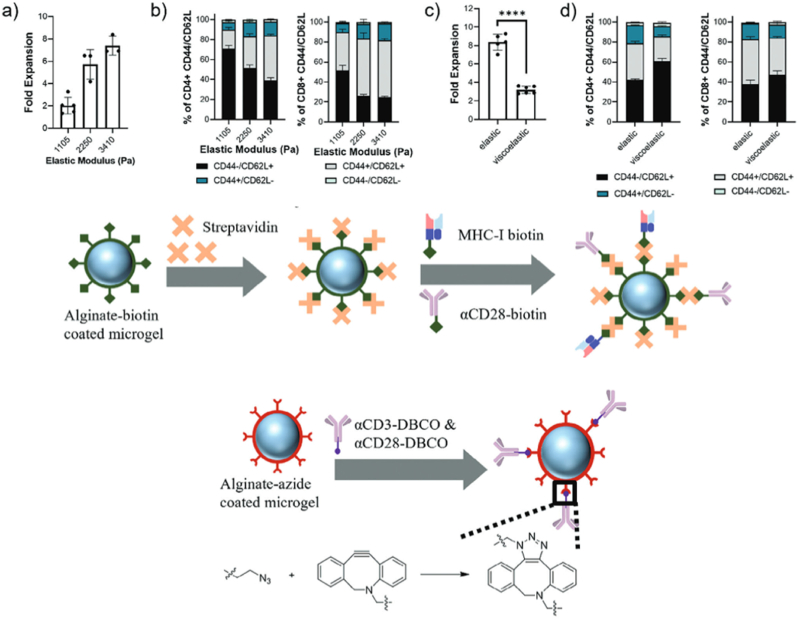
Polyclonal mouse T cell expansion (CD4^+^ and CD8^+^ co-culture) while varying the physical properties of microgels. **a)** Expansion of primarymouse T cells and **b)** CD44/CD62L expression by live CD4^+^ (left) or CD8^+^ (right) T cells that were co-cultured with microgels as a function of stiffnessfor 3 days. **c)** Expansion of primary mouse T cells and **d)** CD44/CD62L expression by live CD4^+^ (left) or CD8^+^ (right) T cells that were co-cultured withelastic or viscoelastic microgels for 3 days. Overall antibody density = 0.4 μg cm−2, *α*CD3/*α*CD28 ratio = 1 in all studies. Reprinted with permission from Ref. [134]. Copyright © 2024 Wiley-VCH GmbH.

ACs can also be used as carriers of antigens like model antigenic peptides to evaluate their antigen delivery capabilities and immunological properties for autoimmune diseases. Surface modifications of ACs have significant potential in the membrane technology field, as it allow specific targets, antigens, and synthetic molecules to control the functions and interactions of these ACs, resulting in customized functionality [132]. The customization of functionality is one of the biggest advantages of such system. As an example, Lou et al. developed microgels with tailored surface biochemical properties to regulate T cell expansion and phenotype, aiming to provide a tunable and modular system for adoptive cell therapy (ACT) of T cells [134]. Surface functionalization of microgels using layer-by-layer coating with oppositely charged polymers allows for efficient and flexible surface-specific conjugation of stimulatory ligands, promoting polyclonal and antigen-specific T-cell expansion. The study found that the concentration, ratio, and distribution of stimulatory ligands, as well as the stiffness and viscoelasticity of the microgels, can regulate T cell expansion and phenotype. Azide-modified alginate is used to coat the surface of microgels, allowing for the subsequent conjugation of DBCO-modified antibodies (such as αCD3 and αCD28) through strain-promoted azide-alkyne cycloaddition (SPAAC). This surface functionalization strategy presents stimulatory ligands on the microgel surface, promoting T cell activation and proliferation. Streptavidin-modified alginate is used for antigen-specific activation and expansion of primary mouse CD8^+^ T cells. The high affinity between biotin and streptavidin allows for the specific binding of streptavidin to the biotin-modified alginate-coated microgels. Biotinylated H-2K(b) MHC class I monomer presenting the SIINFEKL peptide and biotinylated αCD28 are then attached to the surface for antigen-specific activation of OT-1 cells (CD8^+^ T cells whose T-cell receptor recognizes SIINFEKL), as shown in Fig. 10. The mechanical properties of the microgels also impacted T cell activation, with increased elastic moduli leading to increased T cell fold expansion and upregulation of activation markers. The study suggests that antibodies' concentration, ratio, and distribution during T-cell activation significantly affect T-cell phenotype, as shown in Fig. 10.

**Fig. 11:**
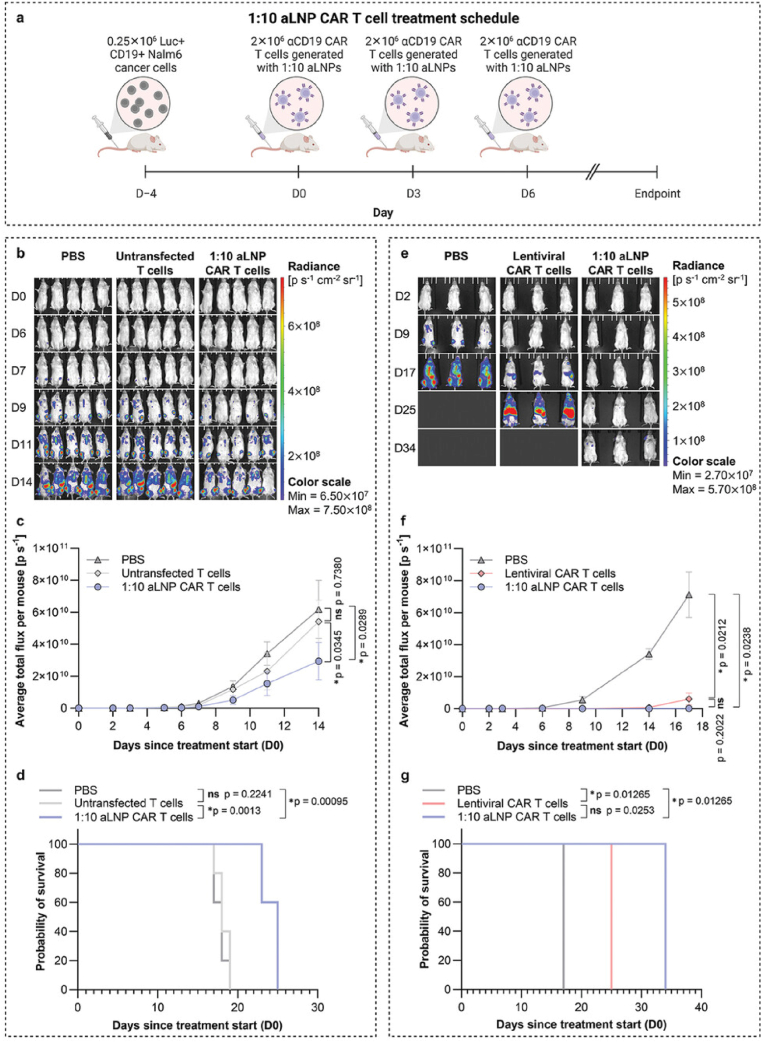
**A.** Adoptive transfer of anti-CD19 CART cells generated with LNPs reduces tumor xenograft mouse model of leukemia. **A.** Schedule used to establish a low-leukemic burden in NSG mice followed by repeated treatments with CAR T cells generated with 1:10 aLNPs. **B.** Time-course IVIS images of Nalm6 (luciferase-expressing human leukemia) tumor-bearing NSG mice treated with three injections of PBS, untransfected T cells, or 1:10 aLNP generated CAR T cells. **C.** Time-course of quantification of average total flux per mouse for the images shown in panel B. Data are presented as mean ± SD. Differences between all treatment means within each day were assessed by a two-way repeated measures ANOVA with post-hoc t-tests using Tukey's correction for multiple comparisons. Only comparisons for day 14 (the final imaging timepoint) are shown. ∗p ≤ 0.05, ns = not significant. **D.** Kaplan–Meier survival curves of the mice following treatment. Differences between survival profiles were assessed using pairwise log-rank tests with Bonferroni corrections for multiple comparisons. To determine significance, the p-values shown (all one-tail) were compared to the Bonferroni-corrected *α* value of 0.0167. ∗ Indicates significance, ns = not significant. (b–d) represents data from a single experiment, for which n = 5 mice per group. **E.** Time-course IVIS images of Nalm6 tumor-bearing NSG mice treated with three injections of 1:10 aLNP generated CAR T cells or PBS, compared to mice treated with a single injection of lentiviral CAR T cells on day 0 (D0). **F.** Time-course of quantification of average total flux per mouse for the images shown in panel (E). Data are presented as mean ± SD. Differences between all treatment means within each day were assessed by a two-way repeated measures ANOVA with post-hoc t-tests using Tukey's correction for multiple comparisons. Only comparisons for day 17 (the last day all mice were alive) are shown. ∗p ≤ 0.05, ns = not significant. **G.** Kaplan–Meier survival curves of the mice following treatment. Differences between survival profiles were assessed using pairwise log-rank tests with Bonferroni corrections for multiple comparisons. To determine significance, the p-values shown (one-tail for PBS *vs.* aLNP and PBS *vs.* lentiviral; two-tail for aLNP vs lentiviral) were compared to the Bonferroni-corrected *α* value of 0.0167. ∗ Indicates significance, ns = not significant. (e–g) represents data from a single experiment, for which n = 3 mice per group. Reprinted with permission from Ref. [136]. Copyright © 2024 Wiley-VCH GmbH.

Using microfluidics to produce gel droplets, Tian et al. presented a microgel with antibodies on the surface capable of continuous paracrine cytokine [135]. The study demonstrated that the microgel leads to a rapid low-differentiation proliferation of T cells *in vitro*. This novel gel droplet simplifies the preparation steps of adoptive cell therapy, improving the therapeutic effect, and providing a new pathway for treating solid tumors. The work also explored the impact of droplet size, quantity of surface antibodies, encapsulated cytokines, and collagen mimetic peptide on T-cell proliferation and anti-tumor ability [135]. Another example with a similar goal, the rapid generation of mRNA CAR (Chimeric Antigen Receptor) T cell cancer immunotherapy was developed by Metzloff et al. [136]. In this work, the ratio of CD3 to CD28 antibody fragments on the LNP surface was adjusted, and a higher proportion of CD28 antibody fragments resulted in increased transfection efficiency and successful *ex vivo* (primary female human T cells procured from the HICat Penn were expanded) cancer cell death (Fig. 11). Additionally, the LNP-generated CAR T cells maintained their cytotoxicity and proliferative ability, indicating their therapeutic potential in Nalm6 acute lymphoblastic leukemia human cells injected into NOD.Cg-Prkdc scid Il2rg tm1Wjl/SzJ (NSG) mice [136].

Sun et al. developed lymphocyte-based homologous targeting artificial antigen-presenting cells (LC-aAPCs) for personalized cancer immunotherapy [40]. This involved the bottom-up assembly of peptide-major histocompatibility complex 1 (pMHC-1) and costimulatory ligands on the surface of lymphocytes. The process begins with acquiring anticoagulant orbital blood from mice, then by separating blood cells and obtaining the lymphocyte layer through density gradient centrifugation. The collected lymphocytes are then incubated with a double-stranded DNA composed of cholesterol and biotin-functionalized DNA, which forms a high-density biotin layer on their surface. Avidin is then assembled on the surface of lymphocytes through specific binding with biotin. Subsequently, pMHCs and aCD28 are assembled onto the lymphocytes through biotin-avidin cross-linking, mimicking the distribution of pMHCs on natural APCs. The engineered LC-aAPCs are capable of effectively expanding antigen-specific T cells *in vitro* and can migrate to peripheral lymphatic organs to activate endogenous antigen-specific T cells *in vivo C57 mice.*

In contrast, lipid NPs (LNPs) are smaller, uniformly sized particles made from lipids, generally ranging from 10 to 200 nm. These particles are often engineered for specific applications like drug delivery or vaccine development. Kim et al. introduced a novel therapeutic vaccine for cancer based on artificial immunogenic cell death lipid NPs (AiLNPs) [137]. Initially, DPPC, DSPE-PEG-NTA(Ni) and cholesterol were dissolved in chloroform. The chloroform was then removed, and the lipids were hydrated with HEPES buffer at 50 °C for 2 h. The hydrated lipids underwent sonication for 10 min to form small liposomes. Subsequently, cancer cell membrane proteins were extracted and inserted into the lipid bilayer by stirring at 50 °C for 1 h. The membrane protein-conjugated liposomes (MPLs) were then extruded 15 times through 200-nm polycarbonate membrane filters at 70 °C to achieve uniform-sized LNPs. Finally, the LNPs were decorated with high-mobility group box 1 protein (HMGB1) and calreticulin (Calr) by mixing at a 10/μl (MPL/protein) ratio and incubating at room temperature for 1 h. HMGB1 acts as an alarmin, signalling tissue damage and inducing inflammation. HMGB1 can bind to toll-like receptors (TLRs), such as TLR2 and TLR4, on antigen-presenting cells (APCs), leading to the activation of the innate immune response. On the cell surface, Calr acts as an "eat-me" signal, promoting phagocytosis by macrophages and dendritic cells. This process is essential for the clearance of dead cells and the initiation of an immune response. Treatment with AiLNPs significantly inhibited the growth of CT-26 tumors in BALB/c mice, as evidenced by reduced tumor size and weight compared to control groups. Besides that, AiLNPs were effective in inhibiting the growth and metastasis of LLC1 tumors in C57BL/6 mice. The AiLNPs induced strong antigen-specific T-cell immunity, with high levels of IFN-γ production in tumor-draining lymph nodes (tdLNs).

**Fig. 12:**
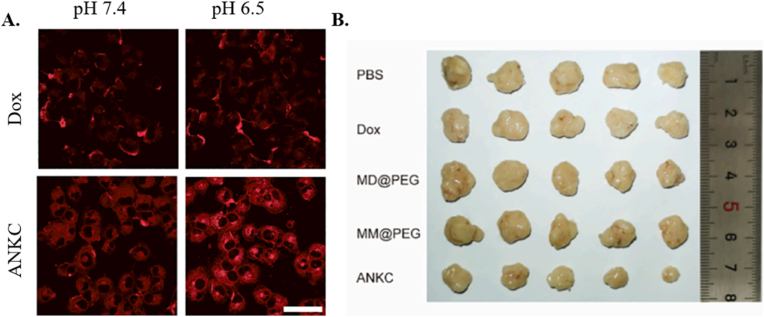
**B.** DOX-resistance MCF-7/Adr cell inhibition taking DOX or ANKC at pH 6.5 (right-side images) or 7.4 (left-side images) performed by confocal imaging and flow cytometry analysis. **D.** Growth inhibition of MCF-7/Adr xenograft tumor in collected tumors Reproduced with permission from Ref. [52]. Copyright © 2024 WILEY-VCH Verlag GmbH.

NK cells can evoke an immune response by detecting and killing tumor cells spontaneously, through perforin-mediated granzyme delivery. Considering this, Li et al. designed a 3D dendritic mesoporous silica NPs (MSNs) functionalized with benzaldehyde as a framework for constructing artificial NK cell (ANKC), as drug-resistance antitumor cells. Melittin, a pore-forming peptide that acts as a perforin initiator, can conjugate to benzaldehyde via a dynamic imine bond under poor alkaline conditions to form acid-sensitive dissociation. The MSNs were then loaded with DOX, an antitumor molecule that mimics the granzymes function. The produced pH-liable ANKC, with the approximate size of 120 nm, was pH-responsive to the slightly acidic environment to control the release of melittin for DOX entry into drug-resistance MCF-7Adr tumor cells. The drug release behavior of ANKC showed that DOX was able to release slowly under slightly acidic conditions, while it retained inside under physiological conditions (pH 7.4). *In vitro* experiments showed increased uptake and retention of DOX by the MCF-7Adr cells after treatment with ANKC, while showing no obvious cytotoxicity (Fig. 12A). Additionally, the MSNs surface was decorated with aminol- and carboxyl-modified PEG to promote their long-circulation ability. *Ex vivo* experiments in MCF-7/Adr xenograft mice comparing free DOX, ANKC, and PEG-decorated MSNs at a dose of 5 mg/kg and intravenous injection, were enriched DOX in tumor in both ANKC and PEG-decorated MSN. As shown in Fig. 12B, *ex vivo* studies confirmed the higher tumor growth inhibitory effect in ANKC-treated mice, with no obvious systemic toxicity. The authors explained this result might be a consequence of combined treatment with DOX and melittin, which lead to increased ATP release and immunogenic cell death (ICD) induction [52].

Innovative approaches in immunotherapy are continually being developed to enhance the immune system's ability to fight cancer. Recent advancements have focused on creating hybrid NPs and cell membrane-coated nanocarriers that modulate immune responses and improve therapeutic outcomes. A proteosome-based ACs in delivering anti-inflammatory microRNA-223 (miR223) to reprogram neutrophils and macrophages, the authors showed that AC cargo delivery modulates leukocyte phenotypes for local immune-mediated pathologies [41]. The use of ACs for miR223 knockdown for driving immune cell suppression through reprogramming was confirmed by upregulation of pro-inflammatory cytokines, including IL1β, interleukin 6 (IL6) and interleukin 12B (IL12B) and phenotype state, performed by Luminex-based multiplex immunoassays in ACs-treated macrophages. To test the effect of phenotype and a change in cancer cell suppression, anti-miR223 ACs were locally injected, and the results indicated that after three injections the pre-neoplastic cells reduced significantly, which was in line with the results of stained sections of cancerous tissue (a significant reduction in local proliferation index). Moreover, the tumor size in zebrafish treated with reprogrammed leukocytes with anti-miR223 ACs remained stable or decreased. Overall, despite the lipid-based ACs, the proteosome-based ACs have longer circulation times and remain more stable, which can prolong their therapeutic effects against anti-inflammation.

## 4 Challenges and translation barriers for ACs in cancer treatment

Each type of AC design has its own translation barriers to overcome. For example, ACs that involve the complexity of biological processes like transcription and translation face major issues with the limiting production of molecules. Nano ACs or biomimetic ACs face similar problems such as NPs [138]. For both, the immunogenicity of ACs is a significant concern as it can lead to adverse immune responses, reducing the efficacy of the treatment. Understanding how immune cells interact with ACs is crucial for developing strategies to mitigate these response. Nowdays, PEGylation has been widely used to extend the circulation time of drugs by attaching PEG chains to therapeutic molecules [139]. However, PEGylation can lead to the formation of anti-PEG antibodies, which reduce its effectiveness and lead to immunogenicity. One of the breakthroughs of NPs and ACs is find alternatives to PEGylation [140]. The first-in-human phase 1 clinical trial with ACs started in 2020 to test hemoglobin vesicles as artificial red blood cells in healthy volunteers. These vesicles are designed to mimic natural red blood cells and serve as an alternative to transfusion. They encapsulate purified and concentrated hemoglobin within PEGylated phospholipid vesicles (liposomes) with a mean particle diameter of 225–285 nm. This design helps to avoid the toxic effects of free hemoglobin and allows the vesicles to function similarly to natural red blood cells in oxygen transport [141]. In the phase 1 trial, healthy male volunteers received a single intravenous injection of HbVs. The routes of administration of ACs also have to be explored according to the application and size of ACs. In tumors, the ACs can be given locally, but when considering intravenous injection, the size and immunoclearance has to be considered. Targeting ACs to solid tumors is challenging due to the complex and heterogeneous nature of the TME. The TME can restrict drug penetration, making it difficult for ACs to reach and effectively treat all cancer cells within a tumor. Therefore, it is necessary to study the interactions between ACs and TME in different tumors.

Besides that, ethical issues related to gene and cell therapy require development of specific regulatory tools that have to evolved with the research progress [142]. There is a lack of long-term biosafety studies on many ACs, which raises concerns about their potential long-term effects on patient.

Scalability and reproducibility are critical for the successful translation of ACs from the lab to clinical settings. However, the field of micro ACs systems is still in its early stages, with most research focused on developing foundational technologies and conducting proof-of-concept experiments [142]. This means that achieving the maturity required for practical applications is still a work in progress. However, it is already evident that integrating synthetic cells with living cells could significantly improve cancer treatments [143]. One of the most notable advancements in this area is CAR-T cell therapy. This therapy involves modifying T cells to better recognize and attack cancer cells by adding a synthetic receptor to these T cells, they can more effectively target and destroy cancer cells, which has already changed the paradigm of cancer treatment [143,144]. Ultimately, the high costs of ACs therapy raises questions about affordability and whether the benefits justify the expense compared to existing treatments.

## 5 Conclusion and future perspectives

It is crucial to select appropriate materials for developing ACs due to their diverse functions, ranging from basic cell models to complex architectures. A commonality among these systems is the need to integrate the lipid bilayer to regulate membrane fluidity, diffusion, and stability, closely mimicking the biological functions of natural cells. Other materials, like polymers, can create more robust membranes with enhanced mechanical resistance. Polymersomes, for instance, can be utilized when durability is essential, as they are more stable than liposome-based vesicles. However, they may lack the natural fluidity and biological functionality of lipid membranes. The use of hydrogels has emerged to provide a matrix for encapsulating components inside ACs. These hydrogels offer a scaffold for controlled release. Inorganic materials like silica, gold, silver, and quantum dots can also be used as internal core of ACs. The unique properties of inorganic materials, such as optical, magnetic, and catalytic characteristics, can add new functionalities to ACs, expanding their potential applications. The main challenge is potential cytotoxicity at high concentrations, clearance and aggregation issues. One solution for the drawbacks of these materials is to combine them in hybrid systems, which are designed to incorporate both membrane functionality and mechanical strength. These systems offer a balance of fluidity, functionality, and durability, enabling them to better mimic natural cell membranes while providing enhanced stability. However, they are more complex to design and optimize.

The right approach for constructing ACs depends on the specific application and desired properties. The top-down approach is suitable for applications requiring precise control over genetic and functional properties, such as creating highly specialized cells for immunotherapy. However, it can be limited by the complexity of natural cells and potential unknown interactions. The bottom-up approach is ideal for constructing novel therapeutic systems with specific engineered functionalities, such as proteinosomes and coacervate microdroplets. The challenge here lies in the complexity of assembling these systems from individual components to the final system. Combining the strengths of both top-down and bottom-up approaches offers high flexibility and the ability to overcome the limitations of each method. This approach is suitable for advanced applications like polymer-lipid membrane nanocarriers and hybrid nanoparticles, but it requires multidisciplinary expertise and careful integration.

Encapsulating small molecules, proteins, and particles inside ACs is possible. The type and characteristics of encapsulated cargo depend on the application and will influence the method of encapsulation chosen. For example, ions and dyes can be incorporated to study permeability, diffusion, and transport mechanisms. Enzymes can be encapsulated to study enzymatic kinetics. DNA and other nucleic acids can also be added to study gene expression as a therapeutic approach. In more complex architectures, liposomes and other organelle-like structures can be used to achieve a more representative model. Despite the challenges associated with encapsulation efficiency, membrane stability, controlled release, scalability, functional integration of membrane proteins, and heterogeneity, ongoing innovations in material selection and encapsulation techniques continue to enhance the capabilities and applications of ACs.

One of the primary advantages of ACs is their versatility in therapeutic and diagnostic applications. They can be constructed to carry specific proteins, deliver drugs to specific sites, and even perform tasks like normal cells, resulting in an endless supply for clinical applications. ACs and biomimetic ACs are customizable and versatile, allowing them to be constructed with custom-made microreactors for specialized functions. For example, in the case of cancer applications can be designed for biosensing, which detects specific molecules, or drug delivery, which releases therapeutic compounds in response to environmental signals. In some cases, biomimetic system can be designed to performed both functions – theranostics.

ACs involve several technical challenges across different methods. While traditional emulsification methods are simpler and more scalable, microfluidics offers superior control and efficiency, making it ideal for applications requiring high precision and uniformity. Each method has its advantages and limitations, affecting the quality, yield, and reproducibility. Overcoming these challenges requires careful attention to specific technical issues and often involves fine-tuning experimental parameters and conditions. Advances in material science and engineering, along with improved methodologies like microfluidics, are enhancing the efficiency, reproducibility, and reliability of ACs [145]. Additionally, both *in vitro* and *in vivo* evaluations of these systems are necessary. Given the complexity of these systems, studying their interactions with the tumor environment is crucial. Furthermore, the complex interactions of ACs with biological systems raise ethical and safety concerns [144]. Overall, mimicking different functions of natural cells is still a significant technical challenge and needs future studies to make further progress in the field [143].

## CRediT authorship contribution statement

**Renata Faria Maia:** Writing – review & editing, Writing – original draft, Methodology, Investigation, Formal analysis, Conceptualization. **Asma Sadat Vaziri:** Writing – review & editing, Writing – original draft, Methodology, Investigation, Formal analysis. **Mohammad-Ali Shahbazi:** Writing – review & editing, Visualization, Validation, Supervision, Conceptualization. **Hélder A. Santos:** Writing – review & editing, Visualization, Validation, Supervision, Resources, Project administration, Funding acquisition, Conceptualization.

## Declaration of competing interest

The authors declare that they have no known competing financial interests or personal relationships that could have appeared to influence the work reported in this paper.

## Data Availability

No data was used for the research described in the article.
